# Time-Dependent Changes in Cortisol and Tympanic Temperature Lateralization During Food Deprivation Stress in Marmoset Monkeys

**DOI:** 10.3389/fnbeh.2020.00123

**Published:** 2020-07-16

**Authors:** Lucas C. Pereira, Rafael S. Maior, Marilia Barros

**Affiliations:** ^1^Primate Center, Institute of Biology, University of Brasilia, Brasilia, Brazil; ^2^Department of Pharmacy, School of Health Sciences, University of Brasilia, Brasilia, Brazil; ^3^Department of Physiological Sciences, Institute of Biology, University of Brasilia, Brasilia, Brazil

**Keywords:** food deprivation, stress, cortisol, tympanic membrane temperature, hemisphere asymmetry, marmoset

## Abstract

Temporal information about food availability can be easily entrained, as in the case of fixed feeding routines of captive animals. A sudden unintentional or deliberate delay (e.g., food deprivation—FD) leads to frustration and psychological stress due to the loss of temporal predictability. How marmosets—an increasingly used small primate—process and respond to FD stress has not been previously assessed. Here we delayed the routine feeding of adult captive marmosets for 3 or 6 h. Blood cortisol concentration was used as a hormonal measure of the stress response. Changes in the left/right baseline tympanic membrane temperature (TMT) were used as an indirect ipsilateral indicator of hemisphere activity. Marmosets that were deprived for 3 h had higher cortisol levels than non-deprived controls. Cortisol concentration in the marmosets deprived for 6 h did not differ from controls possibly due to adaptative mechanisms against the detrimental effects of prolonged high cortisol levels. Interestingly, FD stress may have been processed more symmetrically at first, as indicated by the bilateral increase in TMT at the 3 h interval. As the event progressed (i.e., 6 h), a clear rightward TMT bias suggests that hemisphere activity had become asymmetrical. Therefore, the sudden loss of temporal predictability of an entrained routine feeding schedule induces time-dependent changes in the cortisol stress response and shifts in the TMT (and potentially hemisphere activity) lateralization bias of adult captive marmosets.

## Introduction

Feeding behavior can be influenced by experience-dependent learning processes related to the food item *per se* and its potential rewarding properties, along with its spatial and/or temporal availability (Webb et al., 2009). For example, the latter can be detected when feeding repeatedly occurs at a specific circadian phase. The animal will then likely come to expect or even anticipate the time of day or season when (particular) foods will become available again (reviewed in Mistlberger, 2011). Different foraging strategies may then be adopted at specific times to optimize food intake and minimize energy expenditure (Armstrong, 1980).

The ability to detect, retain, and use such temporal information is frequently demonstrated by animals in captive conditions. Many facilities adopt routine feeding schedules that easily become entrained (Bassett and Buchanan-Smith, 2007). Once this is established, the sudden loss of predictability related to an appetitive event such as feeding can rapidly lead to frustration and psychological stress due to the change in reward contingency (Levine, 2000). This can be the case, for example, during food deprivation (FD) stress; when an animal that can and wants to eat is unable to do so due to the lack of food brought on by events beyond its control (McCue, 2010). As a result, FD can be used to experimentally assess the effects of emotional distress, but it can also inadvertently ensue from routine captive management. Deliberate or unintentional delays in feeding schedules activate the hypothalamus-pituitary-adrenal (HPA) axis and negatively affects the behavior of several species (*chickens*: Beuving et al., 1989; *calves*: Johannesson and Ladewig, 2000; *sheep*: Yayou et al., 2009; Normando et al., 2013; *horses*: Zupan et al., 2020). Although this also seems to be the case for nonhuman primates (NHP), including a rise in cortisol levels, attention so far has been mainly on how they respond to a specific (usually brief) delay (Lado-Abeal et al., 2000; Waitt and Buchanan-Smith, 2001; however see Lyons et al., 2000; Medhamurthy et al., 2007).

In both humans and animals, emotionally-laden events can be asymmetrically processed by the brain. Functional lateralization, established by (epi)genetic factors that interact with environmental cues (Rogers, 2014), allows for higher neural processing capacity, speed, and efficiency (Vallortigara and Rogers, 2005). The right hemisphere seems to preferentially detect, process, and respond to aversive stimuli and negative affect, although there is still some debate over which is the prevailing side (Gainotti, 2012; Rogers, 2009). In NHP, there are several reports of a rightward bias during predatory (*marmosets*: Hook-Costigan and Rogers, 1998; Souza Silva et al., 2007; Pereira et al., 2018), social (*macaques*: Hauser, 1993; Kalin et al., 1998; *chimpanzees*: Parr and Hopkins, 2000; *baboons*: Wallez and Vauclair, 2011), capture/restraint (Tomaz et al., 2003; *marmosets*: Pereira et al., 2019), and novelty-related stress (*marmosets*: Cameron and Rogers, 1999). Then again, Hanbury et al. (2011) reported that neural activity increased bilaterally in restrained bushbabies, whereas lesions restricted to either the left or right hemisphere blunted the response of rhesus monkeys to a snake model (Izquierdo and Murray, 2004). Other studies have also revealed a right-side dominance regardless of the emotional valence (*macaques*: Hauser and Akre, 2001; *chimpanzees*: Fernández-Carriba et al., 2002), or a complete lack of lateralization (e.g., a human intruder with macaques: Izquierdo and Murray, 2004). Hemisphere specialization of emotional activity in NHP thus requires further investigation. Also, to the best of our knowledge, hemispheric processing of FD stress has yet to be addressed.

Here we assessed whether brief FD induces a stress response in adult captive marmoset monkeys and if so, whether it is asymmetrically processed by the brain. For this, we delayed routine food placement for either 3 h or 6 h. Blood cortisol concentration was used as a hormonal measure of the stress response, while a change in tympanic membrane temperature (TMT) was used as an indicator of hemisphere activity. TMT can be a fast, inexpensive, non-invasive, and indirect index of real-time changes in hemisphere activity (Cherbuin and Brinkman, 2007; Propper and Brunyé, 2013), including that of marmosets (Tomaz et al., 2003; Pereira et al., 2018, 2019). It reflects ipsilateral shifts in cerebral blood flow caused by activity-induced changes in brain temperature (Baker et al., 1972; Schiffer et al., 1999; Schuman et al., 1999; Yablonskiy et al., 2000). We hypothesized that FD stress would alter the marmosets’ right TMT and hemisphere activity, yet did not predict the direction of the TMT shift, as this has been shown to increase and decrease in response to stress in this species (Pereira et al., 2018, 2019).

## Materials and Methods

### Ethics Statement

Animal numbers and the procedures herein were approved by the Animal Ethics Committee of the University of Brasilia (no. 006/2017). All procedures were carried out following the Brazilian regulations for the scientific use of laboratory animals (Lei Arouca 11.794/2008), as well as the CONCEA/Brazil and NIH/USA guidelines for the care and use of laboratory animals.

### Subjects, Housing Conditions, and Feeding Routine

Twenty-four adult black tufted-ear marmosets were used (*Callithrix penicillata*; 14:10 males:females aged 4.5–8.0 years old), weighing 335 ± 9 g (295–490 g) at the beginning of the study. They were either born at the Primate Center of the University of Brasilia, being the second or third generation in captivity, or had transferred to this location from other facilities in Brazil at least six years before this study. All subjects were implanted with a subcutaneous radiofrequency transponder identification microchip that had a temperature-recording sensor (BioThermo 985 LifeChip, Destron Fearing, South St. Paul, MN, USA). For more details see Pereira and Barros (2016).

The marmosets used in the study were housed as either same or different-sex pairs in standard home-cages of the same colony room, with both animals from each pair being tested simultaneously. Only pair-housed animals were used to minimize undue stress on the colony. Therefore, there were two male-male pairs due to a limitation in the number of pair-only groups available when the study was held. The housing facility had two parallel rows of 12 cages each (2 × 1 × 2 m; W × L × H), separated by a wire-mesh enclosed central corridor accessed only by husbandry and research personnel. A roof covered the entire central corridor and two-thirds of each home-cage, giving the animals unrestricted access to a protected and uncovered area. Marmosets were exposed to natural light, temperature, and humidity conditions. Each home-cage had a nest-box, ropes, wood perches, a monkey chow dispenser, and a tray for fresh food.

The marmosets were given fresh food once a day, consisting of pieces of fruits and vegetables, boiled eggs, nuts, live mealworms, and/or cooked chicken breast. This diet was prepared daily and individually portioned in food trays for each home-cage. The trays were taken to the colony room where a caretaker immediately placed them, in random order, in their respective home-cages. This procedure usually lasted <5 min and was held on a fairly regular schedule, beginning at 07:00 h (± 10 min). Unconsumed fresh items within the home-cage were removed at 17:00 h. Water and monkey chow were available *ad libitum*. The housing and maintenance conditions complied with the regulations of the Brazilian Institute of Environment and Renewable Natural Resources (IBAMA).

### TMT and SCT Assessment and Analyses

The right and left TMT were assessed with an infrared digital ear thermometer used previously in marmosets (IFR 100 Dual-Mode Thermometer, Microlife, Brazil; Pereira and Barros, 2016; Pereira et al., 2018, 2019). It had an operating temperature range of 10–50^o^C, sensitivity of 0.1 ^o^C, and accuracy of ± 0.2^o^C (between 32.0 and 42.2^o^C). Six readings were made, at 5 s intervals: three in the left ear and three in the right ear. These measurements were taken one at a time, alternating between the two sides. The first ear to be recorded was determined arbitrarily. For each reading, the subject’s external ear was pulled back gently and the thermometer inserted. The device was then activated and the result displayed ca. 1 s later. From the three readings made in each ear, we only used the highest recorded temperature. This was done to minimize a possible error when positioning the thermometer, as the tympanic membrane is hotter than the surrounding tissue (Heusch et al., 2006).

The subcutaneous temperature (SCT) was also recorded to assess whether changes in the TMT were due to an effect of the stress trial on general body temperature. We used a portable universal reader (HS9002B Pocket Reader, Destron Fearing, São Caetano do Sul, Brazil) to display the SCT detected by the subject’s microchip implant (see “Subjects, Housing Conditions, and Feeding Routine” section).

For the SCT and TMT readings, the marmosets were captured with a net and manually restrained. Readings were held immediately before and after the stress trial described below. Disposable probe covers were used in the case of the TMT thermometry. To account for individual variations in the subjects’ initial temperature, a *difference score* was calculated as follows: Δ = post-stress temperature − pre-stress temperature (in °C). Temperatures thus increased during the stress trial when Δ scores were positive, whereas a negative score indicated a decrease in temperature.

### Food-Deprivation (FD) Stress Procedure

The marmosets were randomly assigned to one of four experimental groups (*n* = 6/group). Two groups were food-deprived, receiving their standard diet 3 (FD3h) or 6 h (FD6h) after their normal feeding time. Each of these groups had four males and two females. The other two groups served as controls (CG)–CG3h for the 3 h interval and CG6h for the 6 h interval. Each of these groups had three males and three females. As they were not food-deprived, their standard diet was given at the usual time. Aside from the different feeding times, all subjects were submitted to the same procedure described below. Water remained available *ad libitum* for all four groups. Pair-mates were necessarily assigned to the same experimental group and assessed simultaneously, each one by a different research team.

Each subject was tested only once, but not all members of a group were necessarily tested on the same day. The day before the stress trial was held, all unconsumed food was routinely removed from the home-cage at 17:00 h, along with the chow dispenser. The next day, at 07:00 h (i.e., the usual feeding time), the pair-mates were captured in the home-cage and manually restrained. Their pre-stress SCT and TMT were recorded and the animals returned to the home-cage. Thereafter (i.e., at ca. 07:05 h), pair-mates of the CG groups received their standard diet of fresh food and chow. For pair-mates of the FD groups, these items were withheld for the duration of the pre-established stress interval. During the next 3 h (CG3h and FD3h groups) or 6 h (CG6h and FD6h groups) the subjects remained undisturbed in the home-cage, with or without access to food according to their group. At the end of the pre-established interval, the pair-mates were captured, manually restrained and their post-stress SCT and TMT registered. They were then taken to a procedure room adjacent to the colony facility where a blood sample was taken before being returned to their home-cage. Thereafter, pair-mates of the FD groups received their standard diet; i.e., at ca. 10:05 h for the FD3h group and 13:05 h for the FD6h group. We also recorded the times required to capture each subject and to assess its temperature (Table 1). Behavioral measures were not recorded in this study.

**Table 1:** The time required to perform different steps of the procedure in the monkeys submitted to a single 0 (control), 3- or 6-h food-deprivation stress, expressed as mean ± SEM in seconds.

Parameter	Food-deprivation stress interval			
	3 h		6 h	
	Control	Food-deprived	Control	Food-deprived
*Pre-test procedures*				
Home-cage capture	26 ± 6	38 ± 10	35 ± 10	32 ± 10
Temperature assessment	62 ± 9	68 ± 11	53 ± 21	48 ± 18
*Post-test procedures*				
Home-cage capture	30 ± 7	32 ± 6	39 ± 11	27 ± 10
Temperature assessment	54 ± 7	47 ± 12	43 ± 12	39 ± 12
Blood sampling	136 ± 35	129 ± 24	170 ± 82	181 ± 21

### Blood Sampling and Cortisol Assay

A single blood sample was taken after the post-stress TMT assessment. Pair-mates were sampled simultaneously by the different research teams. Each animal was briefly anesthetized with isoflurane by inhalation using a portable universal vaporizer (Brasmed Vetcase, São Paulo, Brazil) set at 2% and oxygen flow at 1 L/min. Via femoral venipuncture, a 0.5 ml blood sample was taken and placed in a 4 ml vial containing clot activator and serum separator barrier gel (Vacuette, Brazil). After recovery (1–2 min), the animals were returned to the home-cage and monitored during the next 30 min. The duration of the procedure was recorded to assess for possible influence on cortisol levels (Table 1).

Each blood sample was centrifuged at 3,000 rpm for 5 min at room temperature. The serum was transferred to a polypropylene vial and analyzed for cortisol concentration. A single direct chemiluminescence immunoassay (CLIA) was held using a commercial kit for the automated ADVIA Centaur^®^ XP system (Siemens, Brazil). A dilution of 1:50 serum:diluent was used (Multi-diluent 3, Siemens, Brazil). CLIA and dilution parameters were based on a previous study in this species (Pereira et al., 2018). Assay sensitivity was 1 μg/dl, and inter- and intra-assay coefficient of variation from pooled serum were 9.8% and 7.5%, respectively.

### Statistical Analyses

Data were normally distributed and with equal variance, as assessed by Shapiro-Wilk and Levene’s test, respectively. TMT Δ scores were analyzed using a mixed-design two-way analysis of variance (ANOVA), with SCT Δ scores as a covariate, and “group” (CG × FD) and “side” (left × right) as the between-subject and repeated measure variables, respectively. Data from the CG3h and FD3h groups were analyzed separately from the CG6h and FD6h groups. Data on cortisol, body mass, and SCT Δ scores were assessed for between-group and test duration effects *via* two-way ANOVA. Whenever appropriate, subsequent comparisons were performed using Tukey’s test. Pearson correlations were used to establish the relationship between: (1) TMT Δ scores and cortisol concentration; (2) TMT pre- and post-stress temperature values and the respective home-cage capture time; (3) TMT pre- and post-stress temperature values and the respective temperature assessment times; (4) TMT Δ scores and SCT Δ scores; and (5) cortisol concentrations and blood sample times. The significance level for all tests was set at *p* ≤ 0.05.

## Results

**Figure 1:**
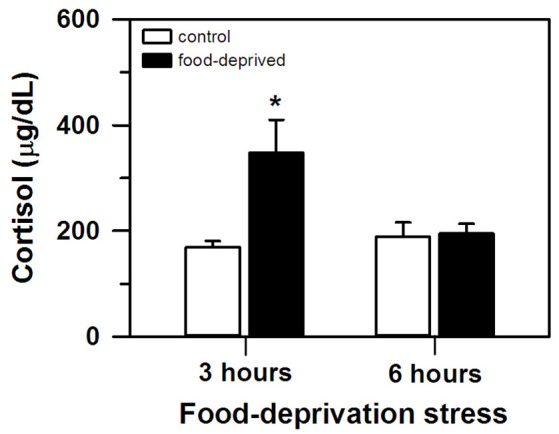
Cortisol concentration (mean + SEM) in the 3- and 6-h food-deprived marmosets, and the non-deprived controls, detected at the end of the experimental trial. *n* = 6/group; *p < 0.05 vs. 3-h control group.

At the end of the stress interval, the marmosets that had been deprived for 3 h had cortisol concentrations that were significantly higher than that of the remaining groups tested (group effect: *F*_(1,20)_ = 6.23, *p* = 0.02; test duration effect: *F*_(1,20)_ = 3.24, *p* = 0.09; interaction: *F*_(1,20)_ = 5.49, *p* = 0.03; Figure 1). The animals that were FD for 6 h and both non-deprived controls had similar cortisol levels that were lower than the FD3h group. Importantly, cortisol concentrations were not related to the time required to obtain the blood samples (CG3h: *r*_(4)_ = 0.60, *p* = 0.21; FD3h: *r*_(4)_ = 0.10, *p* = 0.72; CG6h: *r*_(4)_ = −0.43, *p* = 0.40; FD6h: *r*_(4)_ = −0.48, *p* = 0.35; Table 1).

**Figure 2:**
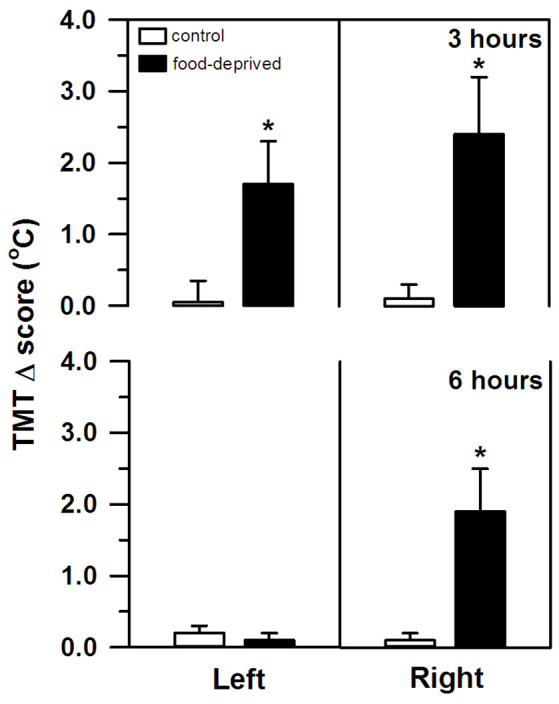
Changes in the left and right tympanic membrane temperatures (TMT, Δ score) of the marmosets that were food-deprived for 3 (top panel) and 6 h (bottom panel) and the respective non-deprived controls. TMT Δ score = post-stress temperature − pre-stress temperature in ^o^C. Data are expressed as mean + SEM; *n* = 6/group. *p < 0.05 food-deprived vs. respective non-deprived control.

TMT, on the other hand, was significantly altered by both stress intervals (Figure 2). However, the specific change seen in each ear differed between the FD3h and FD6h groups. Marmosets that were FD for 3 h had a significant bilateral increase in their TMT. In the corresponding non-deprived controls, we recorded similar temperatures before and after the same 3 h interval in both ears (group effect: *F*_(1,*9*)_ = 38.42, *p* < 0.01; side effect: *F*_(1,*9*)_ = 0.02, *p* = 0.89; interaction: *F*_(1,*9*)_ = 0.24, *p* = 0.64), with no significant effect of the SCT Δ score (*F*_(1,*9*)_ = 2.00, *p* = 0.19). For the marmosets that were FD for 6 h, the right TMT increased significantly, but the left ear was unaffected. In the non-deprived controls, the temperature on either side remained constant during the same 6 h interval (group effect: *F*_(1,*9*)_ = 3.65, *p* = 0.09; side effect: *F*_(1,*9*)_ = 6.54, *p* = 0.03; interaction: *F*_(1,*9*)_ = 6.76, *p* = 0.03; Figure 2). The SCT Δ score did not influence this result as well (*F*_(1,*9*)_ = 0.98, *p* = 0.35).

Correlational analyses did not reveal a relationship between the left or right TMT values and the time required to capture the subjects in their home-cages (*pre-test capture time—*vs. pre-stress left TMT: *r*_(22)_ = −0.06, *p* = 0.77 and vs. pre-stress right TMT: *r*_(22)_ = −0.04, *p* = 0.85; *post-test capture time—*vs. post-stress left TMT: *r*_(22)_ = 0.27, *p* = 0.20 and vs. post-stress right TMT: *r*_(22)_ = 0.07, *p* = 0.75; Table 1). Similarly, TMT values were not associated with the time required to assess the body temperatures (*pre-stress temperature assessment time—*vs. pre-stress left TMT: *r*_(22)_ = 0.61, *p* = 0.77 and vs. pre-stress right TMT: *r*_(22)_ = 0.32, *p* = 0.13; *post-stress temperature assessment time—*vs. post-stress left TMT: *r*_(22)_ = −0.02, *p* = 0.92 and vs. post-stress right TMT: *r*_(22)_ = 0.03, *p* = 0.88; Table 1). TMT Δ scores were found to be unrelated to the cortisol concentrations measured at the end of the stress interval (*left side*: CG3h: *r*_(4)_ = 0.46, *p* = 0.36; FD3h: *r*_(4)_ = −0.26, *p* = 0.62; CG6h: *r*_(4)_ = 0.10, *p* = 0.85; FD6h: *r*_(4)_ = 0.40, *p* = 0.44; *right side*: CG3h: *r*_(4)_ = −0.39, *p* = 0.44; FD3h: *r*_(4)_ = 0.06, *p* = 0.91; CG6h: *r*_(4)_ = −0.60, *p* = 0.21; FD6h: *r*_(4)_ = 0.40, *p* = 0.44), as well as the SCT Δ scores (*SCT Δ score—*vs. left TMT Δ score: *r*_(22)_ = 0.24, *p* = 0.28 and vs. right TMT Δ score: *r*_(22)_ = 0.28, *p* = 0.19). Finally, there were no general effects for group, test duration or interaction in terms of the SCT Δ scores (group effect: *F*_(1,20)_ = 0.03, *p* = 0.87; test duration effect: *F*_(1,20)_ = 0.04, *p* = 0.85; interaction: *F*_(1,20)_ = 0.42, *p* = 0.52; Table 2) or body mass (group effect: *F*_(1,20)_ = 0.33, *p* = 0.57; test duration effect: *F*_(1,20)_ = 1.98, *p* = 0.18; interaction: *F*_(1,20)_ = 3.15, *p* = 0.09; Table 2).

**Table 2:** Body mass and change in subcutaneous temperature (SCT Δ) of the monkeys submitted to a single 0 (control), 3- or 6-h food-deprivation stress, expressed as mean ± SEM.

Parameter	Food-deprivation stress interval			
	3 h		6 h	
	Control	Food-deprived	Control	Food-deprived
Body mass (g)	340 ± 9	318 ± 8	322 ± 13	376 ± 29
SCT Δ score (°C)^a^	0.1 ± 0.1	0.2 ± 0.2	0.2 ± 0.1	0.1 ± 0.1

*^a^Δ score = post-stress temperature − pre-stress temperature*.

### Discussion

In this study, a delay in the routine feeding schedule induced a stress-related reaction in our adult captive marmosets. When food provisioning was withheld for 3 h, circulating cortisol levels were significantly higher compared to non-deprived individuals (FD3h × CG3h group). These control animals received the daily standard diet at the usual time, but like the FD3h group had their cortisol assayed 3 h later. Cortisol concentration was unrelated to blood sampling time and the concentration of non-deprived individuals matched that of non-stressed marmosets (Chrousos et al., 1982; Pryce et al., 2002; Saltzman and Abbott, 2011).

HPA axis hyperactivity has been observed in rhesus macaques due to a short (< 3 h) delay in fixed feeding schedules (Helmreich et al., 1993; Lado-Abeal et al., 2000). Agonistic, self-directed, and stereotyped stress-related behaviors were also seen in stump-tailed macaques when food was given later than expected (< 1 h delay; Waitt and Buchanan-Smith, 2001). Similar results are reported for farm animals (*calves*: Johannesson and Ladewig, 2000; Normando et al., 2013; *chickens*: Beuving et al., 1989; *horses*: Zupan et al., 2020; *sheep*: Yayou et al., 2009). In *ad libitum* fed rodents, corticosterone levels increased only after feeding had been interrupted for ca. 24 h (Galicich et al., 1963; Huang et al., 2016; Johansson et al., 2008; Nowland et al., 2011). Food availability does not usually become entrained under *ad libitum* feeding schedules (Stephan, 2002), and thereby delayed feeding is not expected to immediately induce frustration. However, under experimental settings, switching from a predictable to an unpredictable food reinforcement schedule readily elevated corticosterone concentration (Coe et al., 1983; Davis et al., 1976; Goldman et al., 1973; Levine et al., 1972). This setup may be more akin to the unexpected delay that our marmosets faced. Similar shifts have promptly elevated cortisol levels and negatively affected the behavior of different NHP species (Gottlieb et al., 2013; Lyons et al., 2000; Ulyan et al., 2006; however see Bloomsmith and Lambeth, 1995). When feeding/food reward is an unpredictable event, switching to a predictable schedule (*rats*: Levine et al., 1972) does not affect circulating glucocorticoids. A feeding routine where energy intake varies each day also seems to have a minimal effect on cortisol in NHP (*howler monkeys*: Martínez-Mota et al., 2016). Therefore, a key aspect of our marmosets’ stress-related reaction may have been the sudden loss of temporal predictability related to the entrainment of the routine feeding schedule. However, this should be interpreted with caution as we cannot discard the possibility that cortisol release could partly reflect a homeostatic energy mobilizing response to lower glucose levels due to the FD (McCue, 2010).

The initial rise in circulating cortisol was possibly reversed when the FD interval increased. After a 6 h delay, cortisol levels no longer differed from that of non-deprived controls (FD6 h × CG6h and CG3h). Although this may be an adaptative physiological mechanism of the HPA axis (Habib et al., 2001), other types of stressors have induced a high cortisol response for more prolonged intervals (e.g., *3-week social isolation*: Cinini et al., 2014), so further studies are required. Cortisol release varies according to stress duration (Miller et al., 2007). However, we are presently unsure how FD stress intervals longer than 6 h will affect HPA axis activity. The exact timeframe of this effect may vary between NHP species. In bonnet macaques with a fixed daily feeding schedule, cortisol levels were still elevated ca. 20 h after FD onset (Medhamurthy et al., 2007). Further studies are required for a more comprehensive comparative analysis of the hormonal response of NHP to FD stress.

Also, the marmosets’ TMT rose significantly, but only when the feeding routine was not on-schedule (FD3h and FD6h groups). This was not related to overall body temperature (i.e., SCT), or the time needed to capture the animal or to obtain its temperature. SCT remained unaltered during the procedure and, similar to body mass, did not differ between groups. On the other hand, TMT is an accurate approximation of cerebral temperature (Mariak et al., 1994; Schuman et al., 1999). Variations in baseline TMT seem to correspond to ipsilateral changes in cerebral blood flow and brain temperature (Baker et al., 1972; Schuman et al., 1999; Yablonskiy et al., 2000) induced by shifts in cerebral activity (Schiffer et al., 1999). Given that negative emotional stimuli can increase neuronal activation and regional blood flow (Canli et al., 1998), the rise in our marmosets’ TMT may reflect an ipsilateral increase in hemisphere activity. This is consistent with other between-subject reports in both humans (e.g., Propper and Brunyé, 2013) and NHP (*bushbabies*: Hanbury et al., 2011, 2013; *chimpanzees*: Parr and Hopkins, 2000; *macaques*: Boyce et al., 1996; *marmosets*: Pereira et al., 2019) using TMT measures following different types of stressors (e.g., social interaction, capture/restraint).

It should be noted, though, that TMT increased bilaterally after the 3 h delay, whereas a rightward bias was detected after the longer 6 h interval. Ubiquitous stressful events, such as FD, involve a complex multi-system response controlled by the brain (van Oort et al., 2017). For the appropriate response to occur and at the correct time, the affective, autonomic, neuroendocrine, and cognitive neural networks interact; each having specific functional asymmetries (Gerendai and Halász, 1997; Hagemann et al., 2003; Vogels et al., 1994). As distinct (sub)cortical structures are recruited and/or inactivated at different time lags (Carlson et al., 1988; Raichle et al., 2001), cerebral activity in our marmosets may have shifted over time. The bilateral rise in TMT after the shorter interval may reflect an initial widespread activation pattern. Then again, a prominent bilateral activity may mask a robust right-sided activity, as reported in human infants during social isolation stress (Dawson et al., 1992).

As the stress event progressed in the present study, different areas were possibly inactivated (e.g., lower HPA axis activity reducing cortisol release in the FD6h group), revealing a clear rightward bias in TMT and hemisphere activity. Negative emotional stimuli are frequently associated with dextrally asymmetrical neural activity (reviewed in Gainotti, 2012). A greater right hemisphere bias has been associated with a higher tendency for withdrawal behaviors (*macaques*: Hopkins and Bennett, 1994; Hook-Costigan and Rogers, 1998; *marmosets*: Cameron and Rogers, 1999; *bushbabies*: Hanbury et al., 2013) and more reactive/fearful-like temperament that leads to more intense and/or enduring behavioral reactions (*macaques*: Kalin et al., 1998). Although the shifts in TMT that were detected here were unable to predict the marmosets’ cortisol response to the FD stress, macaques with the greater right than left hemisphere activity had high cortisol concentrations that correlated with a more intense behavioral response toward a human intruder (Kalin et al., 1998). In marmosets, social contact (Rukstalis and French, 2005) and predator mobbing calls (Clara et al., 2008; Cross and Rogers, 2006) have been associated with lower cortisol release, the former being produced with a right hemimouth/left hemisphere bias (Hook-Costigan and Rogers, 1998). Even though we were unable to record the behavioral reactions, these previous studies suggest that hemisphere asymmetry, cortisol release, and behavioral response to stress may be linked, requiring further investigation.

Although cerebral activation during FD stress has yet to be assessed in NHP, time-dependent changes seem to occur for other types of stress. For example, TMT increased bilaterally after a brief restraint event (*bushbabies*: Hanbury et al., 2011), whereas a right-side dominance was seen after longer intervals (*marmosets*: Pereira et al., 2019) or recurring episodes (*marmosets*: Tomaz et al., 2003). On the other hand, brief exposures to a predator model were asymmetrically processed by the right hemisphere (*marmosets*: Hook-Costigan and Rogers, 1998; Souza Silva et al., 2007; Pereira et al., 2018), but the response to a food-retrieval task associated with a predatory stimulus was blunted by unilateral lesions on either side (*macaques*: Izquierdo and Murray, 2004). When processing emotionally-laden stimuli, aspects other than event duration may contribute to the variability in functional hemisphere bias, or even lack thereof, including the type of stimulus (e.g., restraint vs. predatory stress), species (e.g., response to unfamiliar human by marmosets vs. macaques; Izquierdo and Murray, 2004; Pereira et al., 2018) and trait-like characteristics (e.g., fearful temperament; Kalin et al., 1998). It should also be noted that due to the variable and small sample size of each sex in each group, particularly in terms of females, an analysis by sex was not conducted. Sex has been reported to differently influence the cortisol response of male vs. female marmosets (Smith and French, 1997; Cross and Rogers, 2006; Saltzman and Abbott, 2011; Pereira et al., 2018). Caution should thus be taken when generalizing the results. Furthermore, superficial and deep areas in the brain may differ in terms of temperature, heat dissipation mechanisms, and regional blood flow (Sukstanskii and Yablonskiy, 2006). Emotional and autonomic thermoregulatory processes interact bidirectionally (Hagemann et al., 2003) suggesting that aspects unrelated to emotional processing may also affect brain temperature and blood flow (e.g., Hayward and Baker, 1968). Therefore, we are still uncertain as to the exact mechanisms underlying the time-dependent shifts in the TMT and hemisphere activity of our marmosets.

In summary, our results suggest that the sudden loss of temporal predictability of an entrained routine feeding schedule induced a time-dependent neuroendocrine (i.e., cortisol) stress response in adult captive marmosets, as reported for other NHP and non-primate species. This provides important insights into the welfare of captive marmoset colonies, particularly when considering routine management procedures such as food provisioning. Interestingly, this negative emotional event seems to have been processed more symmetrically at first, as indicated by the bilateral increase in TMT at the 3 h interval. As the event progressed (i.e., 6 h), there was a clear rightward TMT bias suggesting that hemisphere activity had become asymmetrical. The exact mechanisms underlying activity-induced changes in brain temperature and blood flow, and how changes in baseline TMT relate to time-dependent shifts in hemisphere activity during aversive events are still unresolved.

### Data Availability Statement

The raw data supporting the conclusions of this article will be made available by the authors, without undue reservation.

### Ethics Statement

The animal study was reviewed and approved by Animal Ethics Committee of the University of Brasilia (no. 006/2017).

### Author Contributions

LP and MB conceived and designed the study, acquired, analyzed and interpreted the data, and drafted and critically revised the manuscript for important intellectual content. RM analyzed and interpreted the data, and drafted and critically revised the manuscript for important intellectual content.

## Conflict of Interest

The authors declare that the research was conducted in the absence of any commercial or financial relationships that could be construed as a potential conflict of interest.
